# Timeliness of recording in the Clinical Practice Research Datalink (CPRD) - an initial step in the implementation of near real-time vaccine safety surveillance

**DOI:** 10.23889/ijpds.v1i1.338

**Published:** 2017-04-19

**Authors:** Andreia Leite, Nick Andrews, Sara Thomas

**Affiliations:** 1 London School of Hygiene and Tropical Medicine; 2 Public Health England

## Objective

Near real-time vaccine safety surveillance (NRTVSS) using electronic health records (EHR) is an option for post-licensure vaccine safety assessment. NRTVSS requires timely recording of outcomes in the database used. Our study aimed to examine recording delays in the Clinical Practice Research Datalink (CPRD) to inform the feasibility of implementing NRTVSS in England using these data. 

## Approach

To examine delays we selected 4 outcomes of interest for NRTVSS: Guillain-Barre syndrome (GBS), Bell's palsy (BP), optic neuritis (ON), and seizures for the period January 2005 to July 2015. Timeliness of CPRD records was assessed in two ways: 1) Using linked CPRD-hospital episode (HES) data to compare the hospital diagnosis date with the date the record was entered in CPRD (system date), 2) Looking at delays in recording (e.g. due to feedback from specialist referral) in stand-alone CPRD. For the latter the event date was compared with the system date. However, system dates can be changed when practice software is updated or there is mass transfer of a patient's records. After investigation, we excluded these uninformative system dates by excluding records from patients who had more than 100 records with the system date on the same day. 

## Results

67813 patients were identified in CPRD (GBS:n=1081, BP:n=15835, ON:n=2236, seizures:n=48866), 64527 in HES (GBS:n=1680, BP:n=8468, ON:n=1746, seizures:n=53080) and 14104 in both databases (GBS:n=356, BP:n=1511, ON:n=226, seizures:n=12036). For the CPRD-HES comparison, 11843 patients with a diagnosis of interest both in CPRD and HES were included (GBS:n=321, BP:n=1374, ON:n=190, seizures:n=9976). Of these, the majority had a record in CPRD before or within 1 month of the HES record (GBS:49.5%, BP:83.8%, ON:66.8%, seizures:69.8%). For 6 months the corresponding percentage was more than 85% for all conditions examined (GBS:85.4%, BP:92.9%, ON:90.0%, seizures:86.6%). For stand-alone CPRD 57317 patients were included (GBS:n=972, BP:n=14275, ON:n=1958, seizures:n=40327). The majority had a record within one month of the event date (GBS:67.9%, BP:89.3%, ON:71.8%, seizures:83%). More than 87% of records occurred within 6 months of the event date (GBS:87.9%, BP:94.4%, ON:91.6%, seizures:94.9%).

## Conclusion

This work shows that most diagnoses examined were recorded with a delay of ≤30 days, making NRTVSS possible. The distribution of the delays was condition-specific and the weekly delay distribution could be used to adjust for delays in the NRTVSS analysis. CPRD can be a viable data source to use in this kind of analysis; next steps will include trial implementation of the system using these data.

